# Clinical Significance of NUDT1 (MTH1) Across Cancer Types

**DOI:** 10.3390/ijms26115137

**Published:** 2025-05-27

**Authors:** Radosław Misiak, Karol Białkowski, Ewelina Dondajewska

**Affiliations:** 1 Department of Medical Biotechnology, Poznan University of Medical Sciences (PUMS), 61-701 Poznan, Poland; 2 Department of Clinical Biochemistry, Ludwik Rydygier Collegium Medicum in Bydgoszcz, Nicolaus Copernicus University in Toruń, 87-100 Bydgoszcz, Poland

**Keywords:** MTH1, NUDT1, oxidative stress, ROS response, prognostic factor, cancer

## Abstract

MTH1 (MutT Homolog 1) protein is one of the enzymes that protect cells from mutagenetic actions of reactive oxygen species. It sanitizes the pool of free nucleotides, making sure that oxidized dNTPs are not incorporated into the DNA. Any misfunction of it would lead to mutations. As such, it has attracted interest of cancer researchers, and multiple studies have been conducted over the years to determine its role in tumor cells. It has been found that MTH1 is not downregulated in most tumor tissues but, to the contrary, often overexpressed. This suggests that MTH1 is used by cancer as an adaptation to increased oxidative stress caused by metabolic reprogramming to support excessive proliferation. Based on this premise, many recent studies have evaluated MTH1 as either prognostic factor, general biomarker or therapeutic target in cancer. Here, we summarize all available research on MTH1 mRNA, protein and its enzymatic activity in clinical samples across various cancer types, identifying a subset of cancers where MTH1 plays an important role. This is particularly evident in cancers characterized by high metabolic activity and oxygen-rich environments, such as hepatocellular carcinoma, renal cell carcinoma, or non-small cell lung adenocarcinoma.

## 1. Introduction

**Figure 1:**
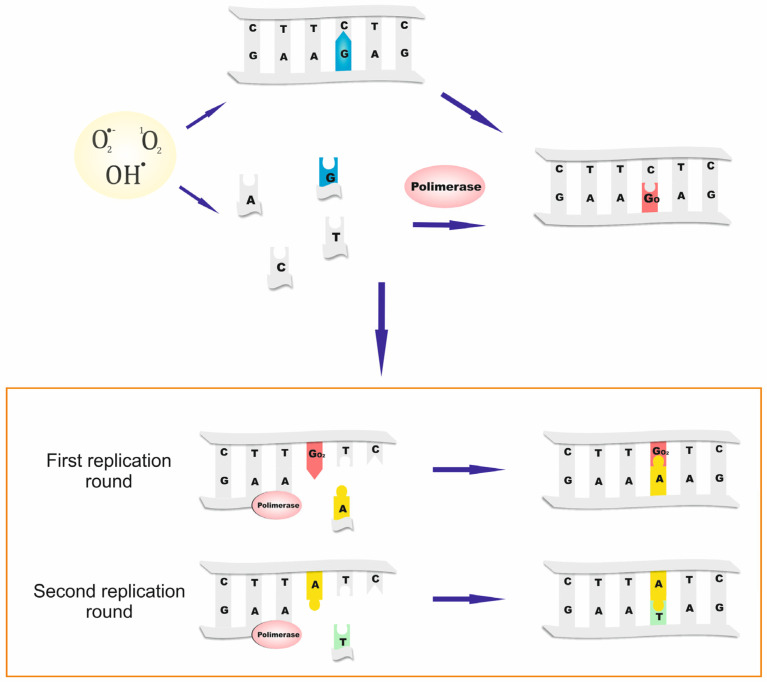
ROS can damage both nucleotides in free nucleotide pool, and nucleobases that are already part of a DNA strand. The nucleotide most prone to oxidation is 2′-deoxyguanosine-5′-triphosphate (dGTP). The oxidized form—8-oxo-dGTP—can be mistakenly incorporated into DNA by polymerase and mispair with adenine, causing permanent mutation after the second replication round.

Reactive oxygen species (ROS) can cause oxidative damage in DNA and the cellular free-nucleotide pools (dNTPs and NTPs). Some of the oxidatively damaged nucleotides, like the 8-oxo-7,8-dihydro-2′-deoxyguanosine-5′-triphosphate (8-oxo-dGTP), can be further incorporated into the DNA during replication, and are capable of ambiguous paring with canonical nucleobases. If these oxidized nucleotides are not removed, they can cause mutations in the subsequent replication rounds. 8-oxo-dGTP can pair with adenine and cytosine, leading to point mutations, in most cases AT→CG. Moreover, the direct formation of the 8-oxo-7,8-dihydroguanine (8-oxo-Gua) in double-stranded DNA may also lead to transversions, mostly GC→TA, which develop after two replication rounds (Figure 1). To prevent an erroneous incorporation of oxidized DNA precursors and subsequent mutations, living organisms are equipped with a system of DNA protection enzymes. One of the main enzymes in this human system is the MTH1 (MutT Homolog 1) protein, coded by the NUDT1 gene [1].

The NUDT1 gene is a human homolog of bacterial mutT that was the first described mutator gene. MutT was discovered by Treffers et al. through research on the spontaneous mutagenesis of *Escherichia coli* that results in acquiring streptomycin resistance [2]. Further studies in the 1970s and 1980s focused on understanding the mechanisms of this mutagenesis in bacteria, and factors which contributed to it. These resulted in an identification of several genes which, when mutated, led to a ‘mutator phenotype’, characterized by an increased frequency of spontaneous mutations [3,4]. The role of MutT as a sanitizing enzyme has been established, and the mechanism by which it prevents the incorporation of the oxidized nucleotides into DNA was described [5]. In 1993, a human cDNA for MutT was cloned by Sakumi et al. and a few years later the general term of Nudix (products of NUDT genes) was established for a class of enzymes hydrolyzing the compounds containing nucleoside diphosphate moiety attached to some X moiety [6,7].

**Figure 2:**
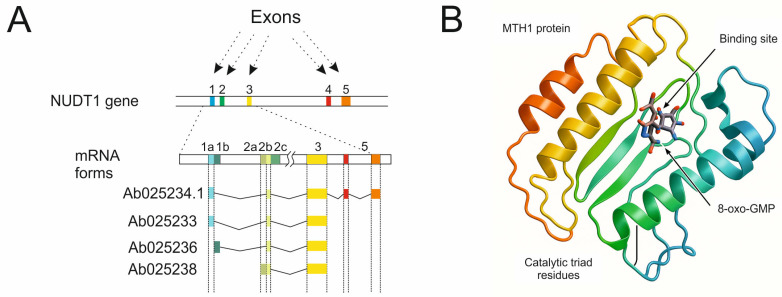
(**A**)—Structure of the NUDT1 gene, consisting of five exons, along with alternatively spliced mRNA variants encoding the most common protein isoform, p18. The numbers of individual splice variants were obtained from the NCBI GenBank database. (**B**)—Three-dimensional structure of the MTH1 protein, highlighting the catalytic site, substrate-binding site, and the molecule representing the primary product of the enzymatic reaction catalyzed by the 8-oxo-dGTPase enzyme.

First of its class, the NUDT1 gene belongs to the NUDIX hydrolase superfamily, which currently consists of 24 known human genes encoding enzymes with phosphohydrolase activity towards nucleoside 5′-triphosphates, dinucleotide polyphosphates, diphosphoinositols, sugar derivatives of ADP and UDP, and even coenzymes such as CoA, FAD, and NAD(P)H [8]. The NUDT1 gene consists of 8924 base pairs and is located on chromosome 7. Several transcript variants exist with transcript 1 being the most common. It encodes the MTH1 protein—a small 156 aa polypeptide with a molecular weight of 17.9 kDa (Figure 2). The main function of MTH1 is preventing the incorporation of ROS-damaged nucleotides into DNA [9,10].

**Figure 3:**
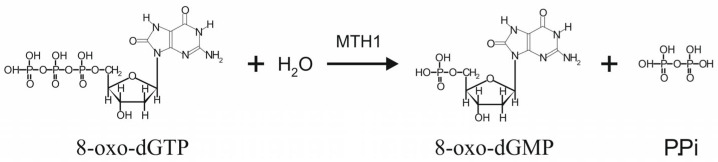
MTH1 protein (8-oxo-dGTPase) breaks down oxidized dGTP nucleotide to 8-oxo-dGMP, which cannot be converted back into the triphosphate form within the cell. Thus, 8-oxo-dGTPase protects cells from incorporating this mutagenic nucleotide into DNA, thereby preventing point mutations scattered throughout the genome and reducing the risk of cancerous transformation.

High concentrations of ROS cause oxidative stress and increase the quantity of oxidative precursors in genomic and mitochondrial DNA, including 8-oxo-dGTP, and to a lesser extent 2-hydroxy-dATP [11]. MTH1 protein is an 8-oxo-dGTPase which degrades these nucleotides to 8-oxo-dGMP, and 2-hydroxy-dAMP, respectively (Figure 3). Mechanistically, the amino acid residues in the phosphohydrolase module of MTH1 create a binding site for 8-oxo-dGTP; the particle is bound by the ionized form of a glutamic acid. At the end of a sequence, a nonionized glutamic acid binds Mg^2+^ cations and a water molecule. The nucleophile attack of a water molecule at the β-phosphate of a substrate at the catalytic site leads to the decomposition of a phosphoanhydride bond, producing a monophosphate. This monophosphate is not re-phosphorylated to form triphosphate in the cellular environment, thereby preventing its use as a DNA building block [12,13].

The question remains, if MTH1 can be a good prognostic factor, general biomarker or therapeutic target for all tumors? Definitely not. As each tissue, and, therefore, each tumor type, differ greatly in the profile of antioxidant enzymes they rely on, so does their dependency on MTH1. Here, we compiled a summary of all available research on clinical samples from a various types of human tumors that show a correlation of MTH1 with clinicopathological features and identified those cancers, for which MTH1 activity was found to be most crucial. These findings support the role of MTH1 as a prognostic factor in some cancers and more importantly validate MTH1 as a target for therapeutic interventions.

## 2. Overexpression of the NUDT1 Gene as a Quantitative Biomarker and a Prognostic Factor in Cancer

**Figure 4:**
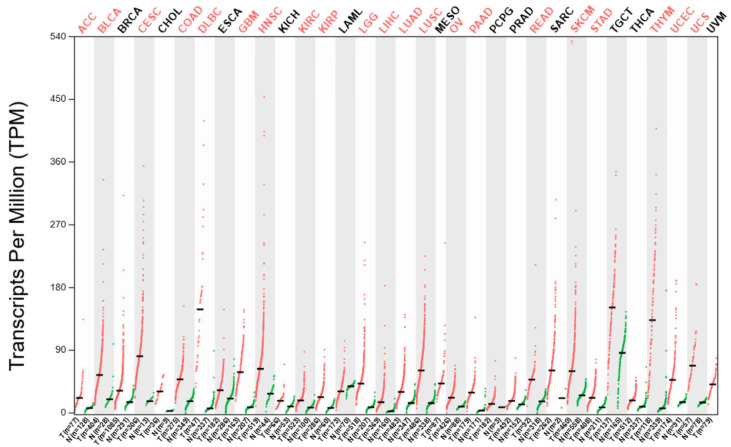
NUDT1 transcript levels were analyzed across 9663 samples of cancer tissues and 5540 samples of normal tissues. Data from the TCGA database were used to generate the chart, and the ANOVA test was selected as the statistical method for result analysis. Red color—cancer tissues; green color—normal tissues. Cancer types in which overexpression was statistically significant compared to reference tissues are marked in red. Abbreviations: ACC—Adenoid Cystic Carcinoma, BLCA—Bladder Urothelial Carcinoma, BRCA—Breast Cancer, CESC—Cervical squamous cell carcinoma and endocervical adenocarcinoma, CHOL—Cholangiocarcinoma, COAD—Colon adenocarcinoma, DLBC—Lymphoid Neoplasm Diffuse Large B-cell Lymphoma, ESCA—Esophageal carcinoma, GBM—Glioblastoma multiforme, HNSC—Head and Neck squamous cell carcinoma, KICH—Kidney Chromophobe, KIRC—Kidney renal clear cell carcinoma, KIRP—Kidney renal papillary cell carcinoma, LAML—Acute Myeloid Leukemia, LIHC—Liver Hepatocellular Carcinoma, LUAD—Lung Adenocarcinoma, LUSC—Lung Squamous Cell Carcinoma, MESO—Mesothelioma, PAAD—Pancreatic Adenocarcinoma, PCPG—Pheochromocytoma and Paraganglioma, OV—Ovarian serous cystadenocarcinoma, PRAD—Prostate Adenocarcinoma, READ—Rectum Adenocarcinoma, SARC—Sarcoma, SKCM—Skin Cutaneous Melanoma, STAD—Stomach Adenocarcinoma, TGCT—Testicular Germ Cell Tumors, THYM—Thymoma, UCEC—Uterine Corpus Endometrial Carcinoma, THCA—Thyroid Carcinoma, UCS—Uterine Carcinosarcoma, UVM—Uveal Melanoma.

**Figure 5:**
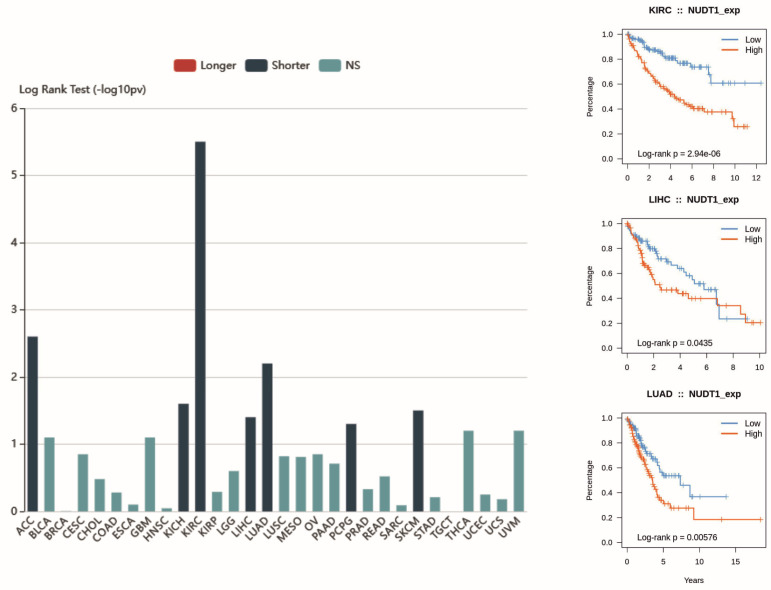
Association between NUDT1 expression and Overall Survival across cancer types. Data from the TISIDB database, Log rank test: *p* < 0.05. Abbreviations: ACC—Adrenocortical carcinoma, BLCA—Bladder Urothelial Carcinoma, BRCA—Breast invasive carcinoma, CESC—Cervical squamous cell carcinoma and endocervical adenocarcinoma, CHOL—Cholangiocarcinoma, COAD—Colon adenocarcinoma, ESCA—Esophageal carcinoma, GBM—Glioblastoma multiforme, HNSC—Head and Neck squamous cell carcinoma, KICH—Kidney Chromophobe, KIRC—Kidney renal clear cell carcinoma, KIRP—Kidney renal papillary cell carcinoma, LGG—Brain Lower Grade Glioma, LIHC—Liver hepatocellular carcinoma, LUAD—Lung adenocarcinoma, LUSC—Lung squamous cell carcinoma, MESO—Mesothelioma, OV—Ovarian serous cystadenocarcinoma, PAAD—Pancreatic adenocarcinoma, PCPG—Pheochromocytoma and Paraganglioma, PRAD—Prostate adenocarcinoma, READ—Rectum adenocarcinoma, SARC—Sarcoma, SKCM—Skin Cutaneous Melanoma, STAD—Stomach adenocarcinoma, TGCT—Testicular Germ Cell Tumors, THCA—Thyroid carcinoma, UCEC—Uterine Corpus Endometrial Carcinoma, UCS—Uterine Carcinosarcoma, UVM—Uveal Melanoma.

Biomarkers play a crucial role in designing tailored treatment plans and closely monitoring patient progress throughout therapy. If treatment outcomes fall short of expectations, adjustments can be made to optimize the approach. The NUTD1 gene has the potential to become a biomarker and specifically a prognostic factor because it is overexpressed in many types of cancer compared to corresponding normal tissues (Figure 4). The impact of this overexpression on patient outcomes is well documented for some cancer types (Figure 5). Furthermore, the main protein product of this gene is easily detectable using various molecular and histopathological methods. Up to now, multiple studies have shown increased levels of mRNA_NUDT1_, MTH1 protein levels or enzymatic activity in tumors, but neither the expression levels nor the expected impact on tumor progression are uniform across different cancer types.

### 2.1. Renal-Cell Carcinoma

The first study of the expression level of NUDT1 in kidney cancers was carried out by Okamoto et al. Tissues obtained from 40 patients were divided into three groups: advanced tumors, tumors in the early stages, and adjacent cancer-free tissues as controls. The overexpression was confirmed in tumors relative to references. Moreover, the overexpression was associated with disease progression as concentrations of mRNA_NUDT1_ increased in advanced tumors relative to tumors in the early stages [14]. Similarly, Wang et al. demonstrated that the level of mRNA_NUDT1_ in postoperative fragments of renal-cell carcinomas collected from 70 patients was statistically higher than in normal kidneys which served as controls [15].

The works of Lin et al. and Xie et al. further show that mRNA_NUDT1_ expression is increased in Renalcell carcinoma (RCC) samples. These results are based on clinical samples, in vitro cell lines, and publicly available database analysis. Having studied 526 of RCC samples from a TCGA database, the authors confirmed that the overexpression of NUDT1 in RCC cancer was correlated with clinicopathological features. The overexpression of 8-oxo-dGTPase is more common in men than in women. Additionally, a higher level of NUDT1 gene expression corresponds to more advanced stages of the disease and is observed across all TNM stages and grades. Results for overall survival were less optimistic for a group of patients with high-level expression of the NUDT1 gene than for a group with low-level expression. Subsequently, a positive correlation between NUDT1 and several genes was observed, including those involved in anti-apoptotic processes (e.g., BCL2L12). Differentially expressed genes (DEGs) showed a dependence on NUDT1, particularly in relation to the humoral immune response, immunoglobulin complex, cytokine-cytokine receptor interaction, and protein digestion. Analyses based on GO and KEGG pathways revealed a correlation between NUDT1 overexpression in RCC and immune infiltration, specifically increased Tregs, CD8+ T cells, follicular helper T cells, and M0 macrophages. Conversely, a negative correlation was noted between NUDT1 expression and immune infiltration involving M1 macrophages, M2 macrophages, resting mast cells, resting memory CD4+ T cells, and monocytes [16]. Further analyses involved two RCC cell lines, 786-O and ACHN, using siRNA to investigate the impact of NUDT1 knockdown on cancer cells. The authors observed reduced viability, migration, and invasiveness in both silenced lines compared to non-silenced controls. Apoptosis increased in the silenced cells, confirming the conclusion that NUDT1 expression in RCC cells is crucial for cancer progression [17].

NUDT1 expression in RCC may be stimulated by factors such as HIF2α, in a common effort to reduce the oxidative stress levels. Shi et al. sought to understand how NUDT1 and HIF2α contribute to ccRCC progression. They examined 34 tissue pairs from RCC patients and three cancer cell lines. In cancer tissues, mRNA_NUDT1_ and MTH1 protein levels were increased compared to normal tissues surrounding the tumor. Similar results were observed for the ccRCC cell lines (A-498, 786-0, Caki-1, and OSRC) when compared to the HK-2 control line. Additionally, the ROC curve analysis for NUDT1 indicated a high potential diagnostic value in ccRCC with AUC = 0.9277 (A high AUC value—close to 1-suggests that NUDT1 can effectively distinguish between RCC and non-RCC cases) [18].

Bioinformatic data analysis of HIF2α knockdown cell lines revealed a positive correlation between oxidative stress and NUDT1 expression, particularly related to mitochondrial formation, division, and nucleotide salvage pathways. To verify this, HIF2α activity was silenced using shRNA, and NUDT1 expression was increased via a lentiviral vector in cancer cell lines. The reduction in proliferation, migration, and invasiveness resulting from HIF2α inhibition was reversed by NUDT1 overexpression. Moreover, NUDT1’s impact on ubiquitination, and therefore stability of SIRT3—an oxidative stress regulator, has been found, underlying a possible mechanism for HIF2α-NUDT1-SIRT3 axis of oxidative stress defense in RCC [18].

Collectively, these findings robustly support the hypothesis that NUDT1 plays a pivotal role in RCC pathogenesis through its involvement in oxidative stress defense, immune regulation, and tumor cell survival mechanisms. The consistent overexpression of NUDT1 across tumor samples and its association with poor prognosis, alongside its regulatory relationship with HIF2α and impact on key cellular pathways, strongly position MTH1 (the protein product of NUDT1) as a promising biomarker candidate for RCC. Nevertheless, further validation in prospective clinical studies is warranted to confirm its clinical utility in diagnosis, prognosis, and potentially in therapeutic targeting.

### 2.2. Hepatocellular Carcinoma

Liver tissues with confirmed hepatocellular carcinoma (HCC) from 23 patients, and adjacent non-cancer samples were collected by Jungst et al. [19]. The authors aimed to study the role of three DNA repair enzymes: MTH1, OGG1, and MYH in HCC. Interestingly, results showed that only mRNA_NUDT1_ levels were elevated in tumors, with a 2-fold increase over surrounding hepatic tissue. Accordingly, levels of 8-oxo-dG were lower in tumor tissue, than in surrounding tissue, possibly due to high activity of MTH1 in tumor. Authors link the increased levels of 8-oxo-dG in surrounding cells with inflammatory infiltration and hepatocellular oxidative damage. The differential expression of DNA damage enzymes is most likely a protective mechanism [19].

Similar results regarding MTH1 levels in HCC were obtained by Zhou et al. In their study, 33 HCC tumor samples were used, healthy adjacent tissues free of cancer served as references. Findings demonstrated an overexpression of mRNA_NUDT1_ in 29 tumors (87.8%), with an average level being two times that of the surrounding tissue. MTH1 protein levels, as measured with IHC, followed the mRNA expression, with a more than 2-fold increase in staining intensity. These results were consistent with in vitro cellular models involving SMMC7721, HepG2, ZIP177 (HCC cell lines) and L02 (healthy liver cell line) tested in two independent studies [20,21].

Hua et al. investigated the level of MTH1 protein in 123 samples of HCC tissues, as well as in adjacent cancer-free tissues and healthy organs from liver transplant donors using IHC [22]. Results indicated overexpression of MTH1 in tumors relative to adjacent non-cancer tissues. They grouped these 123 patients according to the expression of MTH1 protein in HCC tumors: patients with a low expression (*n* = 44), and patients with high expression (*n* = 79). Overall survival after 60 months was much higher for patients with low MTH1 than for patients with high MTH1. Furthermore, the authors detected a correlation between elevated amounts of MTH1 protein and oxidative stress [22].

To confirm these results, authors analyzed 377 pairs of HCC samples and adjacent cancer-free tissues from a TCGA database in terms of mRNA_NUDT1_ levels performed by microarray. They found a statistical significance of overexpression of mRNA_NUDT1_ in HCC samples. Kaplan–Meyer analyses of overall survival were performed. Overall survival in the group with the overexpression of mRNA_NUDT1_ was shorter, relative to patients with a low expression of the NUDT1 gene, confirming results from their study. Results of the TCGA database showed a positive correlation between mRNA_NUDT1_ levels in tissue pairs, relative to the histologic grades and tumor node metastasis [22].

A more extended clinical database analysis of NUDT1 expression across HCC samples was performed by Ou et al. For this purpose, on top of the HCC cases from TCGA database, they used datasets generated by independent research projects available in the Gene Expression Omnibus database: GSE14323 (HCC samples = 55, controls = 60), GSE14520 (HCC samples = 225, controls = 220), GSE41804 (HCC samples = 20, controls = 20), GSE45436 (HCC samples = 93, controls = 41), GSE51401 (HCC samples = 30, controls = 34), GSE62232 (HCC samples = 81, controls = 10), GSE6764 (HCC samples = 35, controls = 40). These experiments were performed with the microarray method to determine the global expression level of the gene. All of them indicated a significant increase in mRNA_NUDT1_ in HCC tissues as compared to controls. Subsequently, the authors confirmed these results with their own study involving 16 pairs of tumor and normal tissues for mRNA_NUDT1_ analysis and 95 pairs of samples for MTH1 protein level assessment by immunohistochemistry. High staining of MTH1 was found in 49/95 tumors (51.6%) relative to controls. NUDT1 levels correlated positively with AFP expression, tumor grade and size and degree of vascular invasion. Overall survival (OS) analysis after 30 months indicated a much higher OS (70%) in the group of patients with low levels of MTH1, as compared to patients with high levels of MTH1 (40%). In vitro studies have shown that the MTH1 protein levels positively correlate with migration, proliferation, survival, and invasion of HCC, as analyzed by silencing NUDT1 using RNA interference in HCC cell lines. It is worth mentioning that endogenous levels of MTH1 protein in these cell lines, were highly variable. The expression levels of MTH1 protein were strongly increased only in the BEL-7402 cells, while in Sk-Hep-1 and Hep-3B cell lines, the levels were decreased as compared to non-cancerous cells. After conducting the GSEA analysis, several pathways related to tumor formation regulated by NUDT1 were identified, including fatty acid metabolism, cell cycle, bile acid and bile salt metabolism, and the PLK1 signaling pathway [23].

To summarize these studies, an overexpression of the NUDT1 gene was observed in the majority of tumor tissue samples obtained from patients. In agreement with that, both bioinformatics analyses and in vitro studies using tumor cell lines also revealed elevated levels of mRNA_NUDT1_. Although MTH1 protein expression exhibited substantial variability, increased levels were detected in most of the analyzed tumor samples. Notably, overall survival was more than 2-fold lower in HCC patients with NUDT1 overexpression compared to those with reduced expression. Furthermore, the involvement of NUDT1 in key metabolic and signaling pathways was confirmed. Collectively, these findings support the potential of NUDT1 as a promising biomarker candidate for hepatocellular carcinoma (HCC).

### 2.3. Colorectal Cancer

Notterman et al. compared the global expression of genes using a microarray test in 18 tumors of the adenoma of the large intestine and adjacent non-cancerous tissues. In 16/18 cases, higher mRNA_NUDT1_ expression was observed in cancer tissues compared to reference tissues [24].

To analyze mRNA_NUDT1_ levels in colorectal cancer (CRC), Qiu et al. obtained 84 pairs of cancer and neighboring cancer-free tissues. Overexpression occurred in 54 out of 84 cancer tissues and a positive correlation was found between tumor size, tumor stage, and mRNA_NUDT1_ expression levels. The abundance of MTH1 protein in CRC samples followed the pattern of its transcript, with elevated levels in 47 out of 84 samples. Interestingly, the levels of MTH1 correlated closely to the levels of Hypoxia-inducible factor 1-alpha (HIF1a), so to better understand the mechanism driving MTH1 overexpression in CRC, authors analyzed the effect of hypoxia on NUDT1 expression in in vitro models. Increased levels of NUDT1 were observed in SW480 and HT-29 lines after 48 and 72 h of hypoxic treatment. Inhibition of HIF1a by siRNA decreased the expression of MTH1, causing the accumulation of 8-oxo-dGTP in both cell lines. These results indicate that MTH1 is regulated by the hypoxic environment of CRC and most likely a direct target of HIF1a [13].

Further studies on clinical samples of CRC include Kumagae et al. who analyzed the levels of MTH1 protein in 56 tissue samples of formalin-fixed and paraffin-embedded CRC (14 cases), dysplasia (9 cases), ulcerative colitis without neoplasm (16 cases), and healthy colon specimens (17 cases). The level of MTH1 protein was much higher in all groups as compared to healthy control, with the highest increase in in colorectal cancer group [25]. Similarly Li et al. analyzed 20 pairs of CRC and neighboring tissues from patients to check the levels of MTH1 protein. The results showed overexpression of MTH1 protein in cancer tissues around 9.4-fold relative to normal tissues. Moreover, it was found that the level of MTH1 protein is related to the AJCC stage and N stage. The authors conducted further observations of patients for 84 months. The overall survival was shorter in the group with MTH1 protein overexpression than in the low-expression group [26].

The enzymatic activity of the MTH1 protein in 10 pairs of CRC tumors and corresponding neighboring tissues was studied by McPherson et al. They used the ARGO method that utilizes an artificial substrate similar to MTH1 and luminescent detection, the same method was used earlier by Ji et al. [27]. To confirm the credibility of the ARGO method, the results of mRNA and protein levels of the NUDT1 gene were also analyzed. RT-qPCR was used to determine the mRNA level, and Western blot was used to determine the MTH1 protein level. The results of each method confirmed the increase in NUDT1 levels in cancer tissues relative to normal tissues. For RT-qPCR, expression increased 2.6-fold, for Western blot 6.1-fold, and for ARGO 5.5-fold. Increased enzymatic activity was detected in 9 cancer samples and was positively correlated with the amounts of MTH1 protein [28].

Bialkowski and Szpila aimed to examine the 8-oxo-dGTPase enzymatic activity in 57 pairs of colorectal cancer and neighboring cancer-free tissues. The method for determining the specific activity of the 8-oxo-dGTPase protein MTH1, developed by Białkowski and Kasprzak, involves ultrafiltration of tissue extracts through regenerated cellulose membranes that retain proteins with a mass over 30 kDa. The 8-oxo-dGTPase activity determination is performed by incubating the extract ultrafiltrate with a reaction mixture containing 8-oxo-dGTP. The reaction is stopped by adding Na_2_EDTA. Then, in the resulting mixture, a quantitative measurement of 8-oxo-dGMP (the enzymatic reaction product) is performed using high-performance liquid chromatography with ultraviolet radiation absorption detection at a wavelength of 295 nm, using 8-oxo-dGMP solutions with known concentrations for calibration. Based on this, the initial 8-oxo-dGTP degradation rate in the mixture is determined and related to the concentration of total protein in the analyzed tissue extract (specific activity). The results showed increased enzymatic activity in 79% of tumors compared to neighboring cancer-free tissues, which corresponded well to the MTH1 protein levels. Unfortunately, while the specificity of this approach to identify CRC tissue is good (83%), the sensitivity of 53% is rather low. Authors followed up on the condition of patients 100 months after resection surgery. Patient data were categorized according to the 8-oxo-dGTPase enzymatic activity in tumors into two groups: over 737.86 U/mg protein (*n* = 32) and lower than 737.86 U/mg protein (*n* = 25). Overall survival in patients with lower activity after 100 months was 71%, while in the second group it was 58%. Patients with MTH1 protein activity in neighboring tissues without cancer were also divided into two groups demonstrating the activities that were higher (*n* = 30) or lower (*n* = 27) than 140.7 U/mg protein. The overall survival in patients with low 8-oxo-dGTPase activity after 100 months of observation was 74% while in the high-activity group it was 52% [29].

In conclusion, NUDT1 gene and protein expression is not uniform across studied colorectal cancer tissues. The enzymatic activity of MTH1 in colorectal cancer as studied by two independent methods and teams and was found to be generally increased. Yet the diversity of CRC, which includes both colon and rectum cancers may require more studies on samples from all histopathological backgrounds to determine MTH1 significance in these malignancies.

### 2.4. Non-Small-Cell Lung Cancer

Research conducted by Chong et al. focused on 70 paired samples from patients with NSCLC tumors (adenocarcinomas, squamous cell carcinomas, and large cell carcinomas) and adjacent cancer-free tissues. The mRNA level of the NUDT1 gene was determined using RT-qPCR and the membrane array method. Increased levels of mRNA_NUDT1_ were detected in 74.3% samples of tumor tissues. Moreover, the authors conducted observations of overall survival over 50 months. Patients with overexpression of the NUDT1 gene had a lower probability of survival than patients with lower expression [30].

Similar associations were confirmed on the protein level by Fujishita et al. Levels of MTH1 protein were assessed by immunohistochemistry on 197 tumor samples from NSCLC patients, and divided into low (86 patients) and high (111 patients) expression. The overall survival with a low MTH1 was statistically higher (92.3%) than in the high MTH1 group (81.6%). Even more striking difference was visible when the disease-free survival was analyzed. It was 83.7% for the first group and only 55% for patients with high levels of MTH1. In further analysis authors used d-ROMs tests and BAP tests on samples from 41 NSCLC patients to check oxidative stress levels and antioxidant capacity in relation to MTH1 protein levels. They found that higher amounts of MTH1 protein were significantly correlated with smokers and high oxidative stress levels, nodal metastases and advanced tumor stage [31].

In another study on NSCLC, Li et al. analyzed the impact of both MTH1 and NUDT5 on tumor growth and patients outcome [32]. 23 patient samples included 19 fragments of adenocarcinoma and 4 samples of squamous cell carcinoma, paired with lung fragments at 10 cm distance from the tumor. Results confirmed higher expression of MTH1 protein in tumor samples. In addition to these findings, authors analyzed MTH1 protein amounts in ten NSCLC cell lines (A549, NCI-H520, NCI-H460, NCI-H1299, NCI-H1975, NCI-H1703, NCI-H661, NCI-H209, NCI-H69 and SK-MES-1), and compared to normal lung cells: CCC-HPF-1 and CCC-HBE-2. A higher level of MTH1 protein was found in all cancer cell lines, relative to reference cell lines. Furthermore, elevated expression of the NUDT1 gene in cell lines was associated with NSCLC metastasis, invasion, and EMT. Authors also analyzed 90 pairs of SCC and 94 pairs of LUAD microarray tissues (TMA). Overexpression of MTH1 protein in TMA was correlated with the T stage, advanced AJCC stage, and N stage [32]. Similarly to Fujishita et al., their overall survival analysis show decreased OS in patients with higher levels of MTH1. The overall survival rate was 70% for patients with low protein levels of 8-oxo-dGTPase, compared to 40% for patients with high levels. NUDT1 knockdown was also performed using shRNA. The absence of this enzymatic protein was associated with increased activity of factors that inhibit proliferation, arrest the cell cycle, and promote apoptosis (such as cyclin D1, P27, Bax, and BCL-2). Another consequence of the lack of MTH1 was the suppression of PI3K/AKT and MAPK signaling pathways and EMT process [31].

One of a few studies to assess the enzymatic activity of MTH1 protein was performed by Speina et al. The authors examined 33 pairs of NSCLC samples and adjacent cancer-free tissues. Authors determined 8-oxo-dGTPase activity and the levels of 8-oxo-2′-deoxyguanosine in DNA. Results showed significantly lower 8-oxo-dG levels and elevated enzymatic activity of 8-oxo-dGTPase in cancer tissues in relation to controls [33]. In several other publications on lung cancer, the overexpression of the NUDT1 gene in cancerous tissues was confirmed. The results were obtained for both mRNA [34] and protein [35,36] levels.

The NUDT1 gene expression levels in the postsurgical sample tissues of patients with non-small cell lung cancers were analyzed in the initial experiment by Patel et al. which showed an increase in mRNA_NUDT1_ in clinical samples of NSCLC. Based on these, authors decided to study the role of MTH1 in KRAS-activated NSCLC by depleting it in the following cell lines: A549-WT, H358, G12S, and H23 using RNA interference. The researchers described two mechanisms for limiting the transformation caused by the KRAS oncogene when MTH1 expression is silenced. In the cells with functional P53, silencing MTH1 triggered oncogene-induced senescence, leading to DNA strand breaks and disruption of proliferation. The alternative mechanism occurred in cells lacking functional P53, such as the H358 and H23 lines. In these cells, no DNA damage was observed, and their proliferative abilities remained intact compared to control cells. Interestingly, a decrease in ROS concentration and a reduction in AKT were observed, which resulted in limiting KRAS expression and preventing proliferation and tumorigenicity effects both in vitro and in vivo [34].

The same team continued research on RAS-dependent transformation in connection with MTH1 expression. In the paper by Giribaldi et al. they focused on HRASV-12 oncogenic transformation. Using Beas2B cell line and shRNA gene silencing, they prove an important role of MTH1 in early tumor development, which leads to an accumulation of high RASV12 expressing cell population in late stage tumors. Moreover, inhibition of MTH1 contributed to suppression of pro-malignant processes such as EMT and glycolytic adaptation. These results suggest that MTH1 inhibitors in clinic, could be a valid treatment option for early stage RAS-dependent lung tumors [36].

The previously mentioned ARGO method was used by McPherson et al. to analyze 12 NSCLC tissue pairs. They found that MTH1 protein activity was elevated in 91% of cancer tissue samples compared to normal tissues. The level of enzymatic activity in healthy tissues ranged from 10.1 to 46.1 pg/μg, whereas in cancer-affected tissues, it ranged from 28.1 to 579.6 pg/μg. The overall fold change in MTH1-specific activity (T/N) was significantly higher in lung tissue at 8.2- compared to 4.8- in pancreas and colon. For lung tissue, the T/N fold change in individual pairs varied from no change (0.77-fold in pair 4) to a maximum of 22.1-fold [28].

To summarize, in non-small cell lung cancer (NSCLC), the overexpression of the NUDT1 gene was detected in the vast majority of analyzed tumor tissues, along with elevated levels of MTH1 protein relative to non-neoplastic samples. High protein expression was also observed across all examined tumor cell lines. A significant correlation was established between NUDT1 overexpression and the presence of histopathological features associated with increased invasiveness in NSCLC. Overall survival was more than 2-fold lower in patients exhibiting NUDT1 overexpression compared to those with lower expression levels. Silencing of the gene encoding the 8-oxo-dGTPase enzyme resulted in the downregulation of signaling pathways involved in cell survival, invasiveness, proliferation, and anti-apoptotic responses. Based on these findings, the MTH1 protein may represent a promising biomarker and therapeutic target for NSCLC.

### 2.5. Breast Cancer

A study conducted by Zhang et al. measured mRNA_NUDT1_ levels in 30 breast cancer samples and 30 matched adjacent cancer-free tissues from female patients. The breast cancer tissues were divided into subtypes: luminal, basal-like, and HER2+. Results showed a more than 3-fold increase in levels of mRNA_NUDT1_ in tumor tissues relative to the controls. Similarly, another cohort of 30 patients’ samples was analyzed for the levels of MTH1 protein using immunohistochemistry. Ten samples of each subtype were studied, and ten samples of tissues surrounding the tumors were randomly taken as controls. Authors found a high level of MTH1 protein in 27/30 tumor samples. By contrast, MTH1 expression in non-cancerous tissues was weak in six patient samples, moderate in one patient sample, and negative in three patient samples. Interestingly, no significant differences in NUDT1 expression between the basal, luminal, and HER2+ subtypes were found on either mRNA or protein levels. MTH1 expression was also not significantly associated with patient age, tumor size, or the grade of lymph node metastasis, contrary to the results obtained for other types of tumors [37].

Another work confirming lack of correlation between NUDT1 expression in breast cancer with the tumor grade or metastatic potential comes from Wani et al. This study shows significantly increased levels of mRNA_NUDT1_ in tumor tissues, with non-detectable levels in normal ductal cells and expression of NUDT1 in 30–80% of cancer cells, but this upregulation was not directly linked to the expression of Cyclin D1, D3, estrogen receptor, p53, ki67 or HER2 [38].

To study the implications of high NUDT1 levels for breast cancer patients’ prognosis, Wright et al. analyzed data from TCGA patients. Patients were divided into two groups according to the level of the NUDT1 gene expression in tumors. Overall survival after 100 months for patients with a low expression of mRNA_NUDT1_ was 75%, and for patients with overexpression—around 55% for patients with a hormone responsive subtype of breast cancer. Interestingly, in a group of patients after resection of receptor negative breast cancers, overall survival was not correlated with the NUDT1 gene expression [39].

Although breast cancers are extensively studied, investigations specifically focusing on the NUDT1 gene represent a relatively small subset. Given that there was not a statistical increase in TCGA samples, and the limited number of other experimental investigations, the MTH1 protein cannot currently be considered a viable biomarker or therapeutic target candidate for breast cancer.

### 2.6. Thyroid Cancer

Arczewska et al. focused on the thyroid tumor tissues from 105 patients. The reference materials were thyroid-adjacent non-cancer tissues. Even though the levels of NUDT1 were very variable between the patients, results showed a significant overexpression of mRNA_NUDT1_ in thyroid tumors, relative to controls. Furthermore, authors analyzed MTH1 protein levels in 21 thyroid tumor specimens (15 PTC, 2 FTC, 2 ATC, 1 FvPTC, and 1 MTC) using IHC and compared them to normal thyroid tissues. Results confirmed high levels of MTH1 in cancer samples, with a predominant cytoplasmic localization. Arczewska et al. studied the correlation between MTH1 and other oxidative stress defense enzymes, and found a strong correlation between MTH1 and GPX1 expression in all studied tissues of thyroid. To further understand this relationship, they depleted MTH1 in several thyroid cell lines, which caused a downregulation of important glutathione-dependent antioxidant defense genes. On top of that, the silencing of MTH1 led to increased ROS levels, defects in proliferation, and DNA damage associated with BSO treatment in thyroid cancer cells [40].

### 2.7. Pancreatic Cancer

Even though TCGA data point to heavy overexpression of NUDT1 in pancreatic tumors, there have not been many studies that specifically focus on MTH1 in this tissue. Notably, pancreatic ductal adenocarcinoma (PDAC) was one of the tumor types analyzed by McPherson et al. using the ARGO method to detect MTH1 activity. The authors analyzed 8 pairs of PDAC tissues and found that MTH1 activity was significantly elevated in 87.5% of the cancerous tissues compared to the control tissues, with the levels ranging from 0.73 to 35.17 pg/μg normal and from 25.13 to 93.82 pg/μg cancer [28].

### 2.8. Esophageal Squamous Cell Carcinoma

Akiyama et al. examined the expression level of NUDT1 in 84 confirmed esophageal squamous cell carcinoma (ESCC) patient samples and corresponding fragments of an adjacent esophageal epithelium with a correct histological structure [41]. Furthermore, they analyzed nine ESCC cell lines (TE1, TE2, TE3, TE5, TE8, TE10, TE12, TE13, and TE15) and three normal fibroblast cell lines from the esophagus (MRC5, BJ, and WI-38). Results show a 4-fold increase in the expression of mRNA_NUDT1_ in cancer tissues and ESCC cell lines compared to healthy tissues and normal fibroblasts. IHC analysis of MTH1 protein expression in these samples confirms the overexpression and links it to deeper tumor invasion, venous invasion, and the more advanced cancer stage. Interestingly, 8-oxo-dG accumulation, which was also analyzed in these samples, was not correlating with any clinicopathological factor.

Akiyama et al., followed up on 52 patients with a low expression of the NUDT1 gene, and 32 patients with a high expression. The 5 year survival percentage after surgery for patients with decreased levels of MTH1 protein was 46.8%. Strikingly, patients with elevated levels of 8-oxo-dGTPase had a 15.2% probability of survival. The disease-free survival rates were 57% and 26%, respectively, highlighting MTH1’s significance as a predictor of ESCC progression and poor prognosis [41].

A very similar study, confirming these results, was conducted by Wang et al. who studied 81 pairs of samples collected from patients with ESCC tumors as well as eight ESCC cell lines from the BNBIO and ATCC. Immunohistochemical findings showed weak detection of MTH1 protein in cancer-free tissues, whereas in tumor tissues, staining varied from weak to strong. In 43 out of 81 tumor samples, a high intensity of staining was detected. The authors found a correlation between the overexpression of MTH1 protein and the malignancy of ESCC. Western blotting analysis showed the overexpression of MTH1 protein in six cancer cell lines (KYSE30, KYSE50, KYSE70, KYSE140, KYSE450, and KYSE520) compared to two human fibroblast cell lines (W138 and IMR90). The authors described the overall survival of 52 patients with low amounts of MTH1 protein and 42 patients with overexpression of 8-oxo-dGTPase over a period of 100 months after tumor resection. Overall survival was more optimistic for patients with low levels of MTH1 protein than for those with the overexpression of the NUDT1 gene. In addition, a positive NUDT1 correlation has been demonstrated with AJCC and T Stages in ESCC tissues. To verify the impact of NUDT1 on ESCC cells, the expression of this enzymatic protein was silenced using shRNA. This led to significant inhibition of proliferation in KYSE50 and KYSE70 cells, effectively slowing down the cell cycle. This conclusion was further supported by elevated levels of P16, P21, and P27 proteins, alongside a significant decrease in Cyclin D. The absence of MTH1 protein also resulted in changes in the MAPK/MEK/ERK signaling pathway. Markers characteristic of mesenchymal cells (MMP7, N-cadherin, and vimentin) were significantly reduced compared to control cells. Conversely, the expression of E-cadherin, which is present in cells with an epithelial phenotype, was increased. Consequently, it was determined that the absence of the 8-oxo-dGTPase limits the proliferation and EMT processes in ESCC cells [42].

Another confirmation of NUDT1 overexpression in esophageal tumors comes from Zhou et al. who, among other cancers, studied ESCC cell lines (EC109, EC9706, and KYSE-450). The HET-1A cell line was used as a control. An increase in mRNA levels was confirmed for all three cancer cell lines, with 1.48-, 1.73-, and 1.60-fold increases compared to the control, proving a mild overexpression [21].

Taken together, several studies show overexpression of MTH1 in ESCC, and associate its elevated levels with poorer patient survival. However, caution is needed when interpreting the general role of MTH1 in esophageal cancers, as data from publicly available sources such as TCGA do not consistently support these findings. This discrepancy may stem from the inclusion of both ESCC and EAC in broader datasets like TCGA, suggesting that the relevance of MTH1 may be specific to the ESCC subtype.

### 2.9. B-Cell Lymphoma

Oksvold et al. conducted research on cell lines covering various types of lymphomas from the LLMPP database and those previously used by Brune et al. The tested types of lymphomas included FL (*n* = 5), BL (*n* = 5), and DLBCL (*n* = 11) and from LLMPP: FL (*n* = 191), ABC-DLBCL (*n* = 176), GCB-DLBCL (*n* = 97), and BL (*n* = 24). Samples from patients in blood banks were used as controls. The results confirmed a high level of mRNA_NUDT1_ expression in GCB DLBCL, BL and ABC cell lines. Further research is needed to assess the significance of this overexpression for the progression of various hematological tumors [43].

### 2.10. Gastric Cancer

Zhou et al. examined 35 pairs of stomach tumor tissue and adjacent non-cancerous tissue. mRNA_NUDT1_ expression was increased around 2-fold in 21 out of 35 cancer tissues relative to the reference tissues. Using immunohistochemistry, authors also measured levels of MTH1 protein in 10 pairs of gastric cancer and neighboring non-cancerous tissues. They have found that the level of MTH1 protein was increased around 1.8-fold in all tested cancerous tissues in relation to control. Furthermore, Zhou et al. tested mRNA and protein levels of the NUDT1 gene in four gastric cell lines (MGC-803, HGC-27, MKN45, and SGC-7901), relative to GES-1, a normal gastric cell line. Increased levels of mRNA_NUDT1_ in all gastric cancer cell lines were observed at about 3-fold compared to the control cell lines. The protein levels were increased around 2.01-, 2.07-, 1.61-, 1.14-folds, respectively, for each cell line in relation to control cell lines [21].

The research of Borrego et al. focused on measuring oxidative stress in gastric cancer patients by assessing various markers, including 8-oxo-dG concentration in tissues, peripheral mononuclear cells (PMNCs) and urine samples from both patients and healthy individuals. Measurements were taken at baseline and one, three, six, nine, and twelve months post-tumor resection. The results showed elevated levels of 8-oxo-dG in cancer tissues compared to normal tissues, which stands in contrast with the results from other tumors, i.e., hepatic. To identify the source of 8-oxo-dG, the expression levels of mRNA_NUDT1_ and other DNA repair enzymes were analyzed, and were found to be increased in tumor tissue. The levels of 8-oxo-dG and other oxidative stress by-products were increased in peripheral mononuclear cells of gastric carcinoma patients compared to healthy subjects. Similar results were found for urine samples, with the diminishing levels of 8-oxo-dG in months following tumor resection. This can be explained by the fact that the efficient functioning of antioxidant enzyme systems, including the MTH1, in managing 8-oxo-dGTP contributes to the high 8-oxo-dG concentration in the intracellular environment. Since its product 8-oxo-dGMP cannot be incorporated into DNA, it accumulates. This, in turn, leads to the excretion of this metabolite, resulting in its elevated concentration in the urine of patients compared to healthy individuals. As a result of their study, authors propose 8-oxo-dG levels in urine as a promising potential gastric carcinoma marker [44].

Regarding the potential role of MTH1 as a biomarker in gastric cancer, although overexpression of mRNA_NUDT1_ and protein has been observed and is in agreement with publicly available database information, more clinical confirmation is needed. Even though the accumulation of 8-oxo-dGTPase and its presence in urine seems to be a promising marker, it cannot be solely attributed to MTH1 activity as other antioxidant enzymes and DNA repair mechanisms also influence the levels of oxidized deoxyguanine, complicating the interpretation of such measurements.

### 2.11. Multiple Myeloma

In their analysis of mRNA_NUDT1_ expression, Zhou et al. examined three multiple myeloma cell lines (U266, RPMI8226, and H929) and 59 samples from CD138+ cells collected from patients with multiple myeloma. The control group consisted of bone marrow samples from healthy donors. The results confirmed that mRNA_NUDT1_ was overexpressed in all three tested cancer cell lines compared to references, and in 27 samples of cancer tissues. In addition, overexpression was significantly higher in tissues with confirmed III ISS stage compared to healthy bone marrow cells. Moreover, increased MTH1 protein levels were detected in each of the cancer cell lines, with the highest amount of MTH1 protein observed in the RPMI8226 line. Further studies are needed to assess the significance of MTH1 expression in multiple myeloma [45].

### 2.12. Osteosarcoma

Moukengue et al. chose osteosarcoma cell lines (HOS-MNNG, KHOS, MG63, U2OS, SJSA-1, G292, CAL72, and 143b) and 13 specimens of osteosarcoma obtained from patients to test the levels of NUDT1. References were mesenchymal stem cells and osteoblasts from healthy donors. In cancer cell lines and cancer tissues, mRNA_NUDT1_ overexpression was confirmed relative to control samples. Changes in expression levels in osteosarcoma tissues ranged from 5 to 30 folds higher compared to mesenchymal stem cells. In cancer cell lines, the mRNA levels of NUDT1 were 3- to 25-fold higher compared to HMSC cells [46].

Another confirmation of the importance of NUDT1 in osteosarcoma development was provided by Qing et al. Their study involved 31 OS tissue samples, 16 normal samples adjacent to the cancer, and OS cell lines (U2OS, MG63, SAOS-2, and MNNG/HOS). MTH1 protein expression was detected in 90.3% of cancer tissues compared to 12.5% in adjacent tissues, as shown by IHC tests. Similar results were obtained from Western blot analysis of the OS cell lines, with lower expression observed in the HFOB osteoblast cell line. Based on this observation, the significance of MTH1 for the survival of osteosarcoma cells was hypothesized. To confirm this, a CCK8 assay was performed on MTH1-knockdown cells to examine proliferation in the cell lines. The silencing was achieved using siRNA, with the control group remaining untreated. The results of the study confirmed the key role that MTH1 plays in the survival of osteosarcoma cell lines in an environment rich in ROS [47].

### 2.13. Oral Squamous Cell Carcinoma

Shen et al. used 31 pairs of fresh oral squamous cell carcinoma (OSCC) tissue samples collected after surgery to analyze MTH1 levels, and found the highly significant overexpression on both mRNA and protein level. Moreover, 62 paraffin embedded samples of OSCC and 18 tissues from healthy individuals, were analyzed by the IHC. A strong positive correlation was found between high levels of NUDT1 and histopathologic tumor grade and tumor stage, as well as poor prognosis of survival. Authors propose NUDT1 as an independent prognostic factor for OS and TSS [48].

### 2.14. Brain Tumors

Iida et al. studied MTH1 levels in 42 neuroepithelial cancers, 5 meningiomas, 2 metastatic brain tumors, and 1 schwannoma. The controls were brain samples from five healthy patients in postmortem examination. MTH1 protein overexpression was detected using immunohistochemistry in the cytoplasm and nucleus of 36 out of 45 cancer tissues. Authors also determined the levels of 8-oxo-dG in these samples by IHC, and they observed an increased amount of this metabolite in cancer tissues, particularly in the cell nucleus, compared to healthy tissues. Both MTH1 levels and 8-oxo-dG accumulation was higher in medulloblastoma and high grade glioma (HGG), suggesting that these undifferentiated brain tumors are exposed to more oxidative stress than differentiated brain tumors [49].

In an experiment conducted by Tu et al., U87MG and U251MG cell lines were used. The cell lines were silenced using shRNA. The effect of hydrogen peroxide on silenced cells was examined and a PI staining revealed that apoptosis occurred more frequently in the knockdown cells compared to the control lines upon H_2_O_2_ treatment. The authors also determined the level of mRNA_NUDT1_ expression in gliomas based on publicly available datasets, and found the mRNA level of NUDT1 was significantly higher in malignant brain tumors than in benign ones [50].

Brain tumor tissues of low and high grade glioma biopsies from 50 patients were thoroughly analyzed by Bhavya et al. for the impact of MTH1 protein. Reference samples consisted of tissues collected from twelve patients after seizures. First, an immunohistochemical examination was conducted to classify gliomas according to their grade of differentiation into high grade gliomas and low grade gliomas (LGG). Secondly, the levels of MTH1 protein in each sample were analyzed by IHC. The results showed an increased level of MTH1 protein in glioma tumor tissues compared to control tissues. However, no correlation was found between the high levels of MTH1 protein and tumor malignancy, (which stands in contrast with previous preliminary works). Authors further measured mRNA_NUDT1_ level, which was increased 1.71-fold for LGG and 1.58-fold for HGG in comparison to cancer-free brain tissues. The overexpression of the MTH1 on the protein level, was higher than its transcripts, as confirmed by both Western blot (2.93 Fc for LGG and 2.84 Fc for HGG) and ELISA (3.84 Fc for LGG and 3.27 Fc for HGG). Using gene silencing and inhibitor treatments on glioma cell lines, Bhavya et al. prove the role of HIF1a in the regulation of MTH1 protein, as well as its impact on cellular DNA damage, apoptosis and ROS generation. In addition, a positive correlation between NUDT1 and factors responsible for angiogenesis (VEGF), as well as invasion and migration (MMP9 and RAC1), was detected. Results of this study suggest a beneficial role of MTH1 inhibitors in treatment of gliomas [51].

Research on brain tumors has primarily focused on quantifying MTH1 protein levels. Elevated expression of MTH1 in LGG and HGG has been reported in a substantial number of studies, in agreement with TCGA transcriptomic data. However, the correlation between elevated gene expression and increased tumor invasiveness or metastatic potential remains questionable. Additional studies are required to reliably determine the diagnostic and prognostic utility of MTH1 as a biomarker in brain tumors.

### 2.15. Malignant Melanoma

Das et al. compared the expression of mRNA_NUDT1_ in malignant melanoma samples from TCGA database with patients’ survival after surgery. The authors stratified patients into two groups: (1) patients with low NUDT1 expression in melanoma (*n* = 401) and (2) patients with overexpression (*n* = 59). The probability of survival for patients with high level mRNA_NUDT1_ was 2 times lower than for the group with low expression of the NUDT1 gene. The same study has shown that inhibition of MTH1 in malignant melanoma, either by inhibitors or gene silencing leads to cytotoxic effects, characterized by mitotic arrest, ROS induction and cell death in most cell lines tested. Similarly to observations made in other cancer types, staining of melanoma clinical samples for MTH1 expression, revealed much higher nuclear presence of MTH1 in stage III-IV melanomas as compared to lower grade tumors and healthy tissues [52,53].

### 2.16. Mesothelioma

Several studies regarding the role of MTH1 in mesothelioma have been conducted by Magkouta et al. They show an interesting correlation between increased levels of MTH1 in tumor cells and increased levels of this protein in tumor endothelial cells. They propose a mechanism in which DNA secreted by tumor cells is detected by endothelial cells and regulates their MTH1 levels and subsequent survival through TLR9-mediated NF-κB signaling, thereby increasing survival, spread, and the formation of new blood vessels for tumors [54]. Besides an increased angiogenesis caused by MTH1 overexpression in mesothelioma models, the authors investigate the correlation of MTH1 levels with immune cell infiltration. Specifically, they focus on TAMs—tumor associated macrophages, and find their MTH1 levels to be elevated. Furthermore, an inhibition of MTH1 is causing an increase in M1 polarization of macrophages, and a common consequence of this is enhanced CD8 T cell activation [55]. Taken together, these data suggest that MTH1 plays an important role in shaping the tumor microenvironment of mesothelioma.

## 3. Small Molecular Inhibitors of MTH1 as a Therapeutic Approach

The overexpression of NUDT1, detected in almost all cancer types studied, indicates an important role for this enzyme in cancer development and progression. Not surprisingly, several therapeutic approaches to target MTH1 protein have been developed. The most successful ones with clinical applications are the small molecular inhibitors based on pyrimidine analogs (Table 1). Their general mode of action relays on binding of the inhibitor to the active site of MTH1 enzyme, rendering it incapable of binding its original substrate, leading to the accumulation of oxidized nucleotides in the cell.

Gad et al. were the first to identify and characterize small-molecule inhibitors of the MTH1 protein. In their study, they demonstrated that the compounds TH287 and TH588 effectively bind to the 8-oxo-dGTPase enzyme in cells. Authors showed the cytotoxic effect of these inhibitors on various cancer cell lines and proved an increase in 8-oxo-dG incorporated into the DNA after the treatment with either TH287 or TH588 in U2OS cells [56]. TH588 showed good efficacy in breast cancer preclinical study (Table 1). A decrease in cell viability of breast cancer cell lines and a reduction in the clone population in test trials relative to control trials were confirmed. In Balb/C-Nu, mice injected with tumor cells and treated with the inhibitor, tumor sizes decreased without any negative effects on liver function or blood biochemical parameters [37]. Since then, first generation inhibitors have been tested in combination with several ROS inducing treatments, such as photodynamic therapy [57] or PEITC [58], and have shown promising results.

Several concerns, however, have been raised in the scientific community, regarding the actual mode of action of TH285 and TH588. It seems that none of the other independently developed MTH1 inhibitors have that significant tumoricidal efficacy. On top of the inconsistency with MTH1 genetic depletion models in different laboratories, this has led authors to question the validity of MTH1 cancer dependency [59]. Samaranayake et al., using the ARGO method to measure 8-oxodGTPase activity in WT and MTH1 depleted cells, found that there is a functionally redundant 8-oxodGTPase activity that is not diminished by MTH1 depletion or MTH1 inhibitors. These findings suggest that MTH1 contribution to general 8-oxodGTPase activity varies across cancer cells and MTH1 independent compensatory mechanisms must exist. On top of TH287 and TH588, authors tested three other inhibitors (IACS, AZ-21, BAY-707) that caused similar decrease in overall 8-oxodGTPase activity, but only TH287 and TH588 reduced cancer cell viability [11]. Gul et al. decided to investigate whether other mechanisms might underlie the cytotoxicity of TH588 inhibitor using CRISPR screening. They discovered pathways and complexes responsible for regulating the kinetochore spindle during mitosis, which may be indirect or direct targets of TH588. The inactivation of spindle regulation by TH588 leads to the selection of cancer cells that use alternative pathways to carry out correct mitotic divisions. A mechanism was identified that prevents cancer cells sensitive to the inhibitor from resuming the cell cycle after being arrested in the G1 phase. However, the CRISPR analysis only included targets within the cell cycle and kinases; the broad genome libraries were not analyzed, which could potentially expand the range of targets for this first-generation inhibitor [60].

The most promising MTH1 inhibitor was introduced in 2016 by Berglund et al. as a second generation inhibitor—TH1579 (Karonudib). It is an analog of TH588 with better potency and selectiveness, good oral availability and anticancer effectiveness, as demonstrated using melanoma patient-derived xenografts, and colon cancer SW480 and HCT116 xenografts [61]. In line with their hypotheses, that MTH1 inhibitors exert highest tumoricidal properties in cancers with high levels of ROS, the same team has studied the effects of TH1579 on glioblastoma cells in vitro and in a zebrafish model. Results show that the high oxidative pressure of GBM is indeed making these cancer cells vulnerable to toxic effects of TH1579, even in treatment resistant models [62]. Beyond testing the activity of TH1579 on another type of cancer, this research focused on comparing it with another class of inhibitors, such as BRAF, in Cutaneous Malignant Melanoma (CMM). As a result, treatment with BRAF inhibitors is only about 50% effective. The combination of both inhibitors yielded satisfactory results in both in vitro and in vivo studies [52]. Another approach to verify the effectiveness of Karonudib, relative to the first-generation inhibitor TH588, was performed on osteosarcoma. Both small-molecule inhibitors caused the arrest of cell cycle in osteosarcoma cell lines. Furthermore, the potential drug significantly increased the level of apoptosis in cancer cells compared to control samples by increasing the activity of the caspase 3/7 pathway. Analysis of the level of γH2AX expression confirmed the increased frequency of double-strand breaks in the DNA chain [46]. Even more striking results have been obtained for hepatocellular carcinoma. TH1579 caused chromosome aberrations and alterations in the mitotic cycle, both of which made cancer cells unable to sustain their proliferation. In vivo tests conducted on mice for 6 weeks showed that Karonudib stopped HCC tumor growth. The authors also observed a loss of body weight and a decrease in tumor progression in the mice treated with the Karonudib [22]. Hematological tumors were another group of cancers on which the effectiveness of Karonudib was tested (Table 1). The authors selected several leukemia cell lines. Additionally, they obtained freshly removed biopsy samples from AML patients. High drug tolerance with the potential for oral administration is a particularly desirable feature, given the limited AML treatment options that do not require hospitalization. This second-generation inhibitor kills not only AML cells but also leukemic stem cells (LSCs) that are responsible for the formation of partially differentiated leukemia blast cells, and thus for significant disease progression [63]. The previous study focused primarily on the action of Karonudib in relation to leukemia, considering only one lymphoma cell line. It was confirmed that the inhibition of the MTH1 protein in lymphomas with TH1579 leads to apoptosis through the incorporation of oxidized dNTPs and cell cycle arrest. Like in the previous test, the regrowth of tumors occurred in the absence of TH1579 [43]. The overall mechanism of action of Karonudib was established as a dual mechanism: firstly, by direct inhibition of MTH1, which causes increased incorporation of toxic nucleotides into DNA, and secondly, by inhibition of microtubule dynamics causing disturbed chromosomes alignment and cell cycle arrest [43,63,64].

Karonudib is in the phase I/II clinical studies, with a completed dose escalation study and currently recruiting patients for combination study with Idarubicin for the treatment of relapsed or refractory Acute Myeloid Leukemia or high risk Myelodysplastic Syndrome [55,64].

**Table 1:** MTH1 inhibitors across tested cancer types.

Inhibitors	Authors	Cancers	IC50
TH287	Abbas et al. [59]	NSCLC	N
	Gad et al. [56]	Colorectal cancer	0.8 nmol/L
	Gad et al. [56]	Breast cancer	
	Gad et al. [56]	Melanoma	
	Huang et al. [57]	Osteosarcoma	0.9 nmol/L (with DOX)
TH588	Zhou W et al. [21]	Gastric cancer	10.23 nM
	Moukengue et al. [46]	Osteosarcoma	4.48–17.37 μmol/L
	Gad et al. [56]	Colorectal cancer	5.0 nmol/L
	Pompsch et al. [65]		5 mM
	Van der Waals et al. [66]		N
	Gad et al. [56]	Breast cancer	5.0 nmol/L
	Zhang et al. [37]		9.78, 6.96 and 8.97 μM
	Gad et al. [56]	Melanoma	5.0 nmol/L
	Berglund et al. [61]		N
	Pudelko et al. [62]	Glioma	12.1 µM
	Ikerjiri et al. [58]	Lymphoma	5 nM
	Ikerjiri et al. [58]	Leukemia	5 nM
TH1579	Hua et al. [22]	Hepatocellular carcinoma	N
	Oksvold et al. [43]	Lymphomas (DLBCL, Burkitt’s)	0.1–0.3 μM
	Moukengue et al. [46]	Osteosarcoma	0.31–16.26 μmol/L
	Pudelko et al. [62]	Glioma	1.1 μM
	Berglund et al. [61]	Melanoma	N
	Berglund et al. [61]	Colorectal cancer	N
	Magkouta et al. [55]	Mesothelioma	N
	Sanjiv et al. [63]	Leukemia	417.4 nmol/L
(S)-Crizotinib	Ji et al. [67]	Gastric cancer	21.33 and 24.81 μM
Echinacoside	Zou et al. [68]	Hepatocarcinoma	7.01 μmol/L
		Osteosarcoma	
		Breast cancer	
		Colorectal cancer	
Mi-743	Zhou et al. [21]	Gastric cancer	91.44 ± 1.45 nM

## 4. Conclusions

Many, but not all tested tumor tissues are characterized by the upregulation of MTH1. Clear overexpression is seen in tumors that have metabolically active oxidative environments, such as hepatocellular carcinoma, renal cell carcinoma or non-small cell lung adenocarcinoma, while in some malignancies, such as multiple myeloma, fewer than half cancerous cells were showing MTH1 overexpression. Furthermore, for the most tumors a correlation between high levels of MTH1 protein and higher tumor grade can be established, but importantly in some, such as breast cancer or brain malignancies, no such correlation was found. Caution is warranted when evaluating the role of MTH1 in cancers such as PDAC, thyroid cancer or specific hematological malignancies, where more research is needed to fully understand the impact of MTH1 on these tumors’ progression. Regarding the potential use of MTH1 expression levels as a biomarker, research shows, that even if the difference between cancerous and neighboring tissue levels of MTH1 is statistically significant across analyzed samples, it is not enough to be indicative of cancerous state. A notable exception would be RCC. The overlap in mRNA_NUDT1_ or MTH1 protein levels between tumor and normal tissues in most other cancer types makes it challenging to establish a specific threshold that could reliably distinguish cancerous tissue. In light of these observations, qualifying MTH1 as a definitive cancer marker may be challenging to say the least. However, it could complement other currently used markers, such as Ki67 or CEA proteins. On the other hand, it is a valid strategy to use MTH1 levels as a prognostic factor in some types of tumors, especially liver, kidney, lung and some head and neck cancers. Researchers are in agreement that high levels of MTH1 protein in these malignancies lead to worse outcomes post tumor resection. For this group of patients, additional treatment options, more frequent check-ups, and the potential implementation of MTH1 inhibitors could be considered. Karonudib (TH1579) warrants special attention, as it is currently a primary focus in ongoing clinical trials.

## Abbreviations

The following abbreviations are used in this manuscript:

8-oxo-Gua	8-oxo-7,8-dihydroguanine
8-oxo-dGTP	8-oxo-7,8-dihydro-2′-deoxyguanosine 5′-triphosphate
ACC	Adenoid Cystic Carcinoma
BLCA	Bladder Urothelial Carcinoma
BRCA	Breast invasive carcinoma
CESC	Cervical squamous cell carcinoma and endocervical adenocarcinoma
CHOL	Cholangiocarcinoma
CMM	Cutaneous Malignant Melanoma
COAD	Colon adenocarcinoma
CRC	Colorectal cancer
DEGs	Differentially expressed genes
dGTP	2′-deoxyguanosine 5′-triphosphate
DLBC	Lymphoid Neoplasm Diffuse Large B-cell Lymphoma
dNTPs, NTPs	2′-deoxyribonucleoside 5′-triphosphates, ribonucleoside 5′-triphosphates
ESCA	Esophageal carcinoma
ESCC	Esophageal squamous cell carcinoma
GBM	Glioblastoma multiforme
HCC	Hepatocellular carcinoma
HGG	High grade gliomas
HIF1a	Hypoxia-inducible factor 1-alpha
HNSC	Head and Neck squamous cell carcinoma
KICH	Kidney Chromophobe
KIRC	Kidney renal clear cell carcinoma
KIRP	Kidney renal papillary cell carcinoma
LAML	Acute Myeloid Leukemia
LGG	Low grade gliomas
LIHC	Liver Hepatocellular Carcinoma
LSCs	Leukemic Stem Cells
LUAD	Lung Adenocarcinoma
LUSC	Lung Squamous Cell Carcinoma
MESO	Mesothelioma
MTH1	MutT Homolog 1
NUDT1	Nudix Hydrolase 1
OS	Overall survival
OSCC	Oral squamous cell carcinoma
OV	Ovarian serous cystadenocarcinoma
PAAD	Pancreatic Adenocarcinoma
PCPG	Pheochromocytoma and Paraganglioma
PDAC	Pancreatic ductal adenocarcinoma
PMNCs	Peripheral mononuclear cells
PRAD	Prostate Adenocarcinoma
RCC	Renal-cell carcinoma
READ	Rectum Adenocarcinoma
ROS	Reactive oxygen species
SARC	Sarcoma
SKCM	Skin Cutaneous Melanoma
STAD	Stomach Adenocarcinoma
TGCT	Testicular Germ Cell Tumors
THCA	Thyroid Carcinoma
THYM	Thymoma
UCEC	Uterine Corpus Endometrial Carcinoma
UCS	Uterine Carcinosarcoma
UVM	Uveal Melanoma
VEGF	Factors responsible for angiogenesis
